# First case report of a *NUP98-PMX1* rearrangement in de novo acute myeloid leukemia and literature review

**DOI:** 10.1186/s12920-021-00979-y

**Published:** 2021-05-17

**Authors:** Weijia Fu, Aijie Huang, Hui Cheng, Yanrong Luo, Lei Gao, Gusheng Tang, Jianmin Yang, Jianmin Wang, Xiong Ni

**Affiliations:** Department of Hematology, Institute of Hematology, Changhai Hospital, 168 Changhai Road, Shanghai, 200433 China

**Keywords:** De novo, Acute myeloid leukemia, Case report, *NUP98-PMX1*

## Abstract

**Background:**

The nucleoporin 98 (*NUP98*)-paired related homeobox 1 (*PMX1*) fusion gene, which results from t(1;11)(q23;p15), is rare in patients with acute myeloid leukemia (AML). Currently, only two cases of chronic myeloid leukemia in the accelerated phase or blast crisis and three cases of therapy-related AML have been reported. Here, we first report a patient with de novo AML carrying the *NUP98-PMX1* fusion gene.

**Case presentation:**

A 49-year-old man diagnosed with AML presented the karyotype 46,XY,t(1;11)(q23;p15)[20] in bone marrow (BM) cells. Fluorescence in situ hybridization analysis using dual-color break-apart probes showed the typical signal pattern. Reverse transcription-polymerase chain reaction (RT-PCR) analysis suggested the presence of the *NUP98-PMX1* fusion transcript. The patient received idarubicin and cytarabine as induction chemotherapy. After 3 weeks, the BM aspirate showed complete remission, and the RT-PCR result for the *NUP98-PMX1* fusion gene was negative. Subsequently, the patient received three cycles of high-dose Ara-c as consolidation chemotherapy, after which he underwent partially matched (human leukocyte antigen–DP locus mismatch) unrelated allogeneic hematopoietic stem cell transplantation (HSCT). The follow-up period ended on September 30, 2020 (6 months after HSCT), and the patient exhibited no recurrence or transplantation-related complications.

**Conclusion:**

This is the first report of a patient with de novo AML carrying the *NUP98-PMX1* fusion gene. The reported case may contribute to a more comprehensive profile of the *NUP98-PMX1* rearrangement, but mechanistic studies are warranted to fully understand the role of this fusion gene in leukemia pathogenesis.

**Supplementary Information:**

The online version contains supplementary material available at 10.1186/s12920-021-00979-y.

## Background

The nucleoporin 98 (*NUP98*) gene encodes a 98 kDa protein and is located on chromosome 11p15. NUP98 is part of the nuclear pore complex and regulates the transport of proteins between the cytoplasm and nucleus. Structurally, the N-terminus of NUP98 contains numerous phenylalanine-glycine (FG) and glycine-leucine-phenylalanine-glycine (GLFG) repeats. The first and third functional domains consist of FG and GLFG repeats, respectively. The second domain contains the Gle2-binding sequence, which is the binding site of the RNA export factor RAE1, and the fourth domain is the RNA-binding site [1–3]. Translocations involving the *NUP98* gene are rare but play important roles in the initiation and development of hematopoietic malignancies. To date, the *NUP98* gene has been implicated in hematopoietic development and found to fuse with more than 30 partner genes and contribute to the onset of leukemia [4]. These partner genes can be separated into two categories, namely homeobox (*HOX*) and non-homeobox (non-*HOX*) genes. *HOX* genes represent a class of transcription factors that share a conserved DNA-binding motif called homeodomain (HD) and include seven clustered “class I” *HOX* genes (*HOXA9*, *HOXA11*, *HOXA13*, *HOXC11*, *HOXC13*, *HOXD11*, and *HOXD13*) and five non-clustered “class II” *HOX* genes (*HHEX*, *GSX2*, *PRRX1*, *PRRX2*, and *POU1F1*). Non-*HOX* genes share a coiled-coil domain and include multiple genes (*DDX10*, *TOP1*, and *NSD1*).

The rearrangement of t(1;11)(q23;p15) involving *NUP98* and the class II *HOX* gene paired related homeobox 1 (*PMX1*) has been rarely reported thus far. To date, only five cases of *NUP98-PMX1* fusion have been reported, involving chronic myeloid leukemia in the accelerated phase, blast crisis, or therapy-related acute myeloid leukemia (AML) (Table 1) [5–8]; no case of de novo AML carrying the *NUP98-PMX1* fusion gene has been reported. Furthermore, there is insufficient information regarding the clinical features, appropriate treatment, and outcomes of patients with the *NUP98-PMX1* fusion gene.

**Table 1:** Summary of six cases of leukemia with *NUP98-PMX1* rearrangement

Case no	Year	Sex	Age (years)	Primary diagnosis	Current diagnosis	Karyotype	Treatment	Outcome	References
1	1999	M	55	NHL stage III, gastric cancer	t-AML	46,XY,t(1;11)(q23;p15)	Topo II inhibitor; MACOP-B	Dead	[5]
2	2007	M	74	Liposarcoma	t-MDS/t-AML	46,XY,t(1;11)(q23;p15)[17]/46,XY[3]	Doxorubicin, ifosfamide, radiation	Dead	[6]
3	2004	M	42	CML-AP	CML-AP	46,XY,t(1;11)(q21;p15),t(9; 22)(q34;q11)[30]	Hydrea, Myleran	Dead	[7]
4	2004	F	44	Breast carcinoma	t-AML	46,XX,t(1;11)(q25;p15)[17]/46,XX[3]	Adriamycin, Cytoxan, 5-FU, BMT	Dead	[7]
5	2006	M	51	CML-CP	AML (CML BP)	46,XY,t(1;11)(q23;p15),t(9; 22)(q34;q11)	Hydrea; arabinosylcytosine, aclamycin, daunorubicin	–	[8]
6	2020	M	49	De novo AML	De novo AML	46,XY,t(1;11)(q23;p15)[20]	Idarubicin, cytarabine, BMT	Alive	Current report

*NHL* non-Hodgkin lymphoma, *CML* chronic myelogenous leukemia, *AP* accelerated phase, *CP* chronic phase, *AML* acute myeloid leukemia *t-AML* treatment-related acute myeloid leukemia, *MDS* myelodysplastic syndrome, *BP* blast phase, *MACOP-B* methotrexate with doxorubicin, cyclophosphamide, vincristine, prednisone, and bleomycin, *5-FU* 5-fluorouracil, *BMT* bone marrow transplant

This is the first report of de novo AML in a patient carrying the *NUP98-PMX1* fusion gene. For a comprehensive understanding of this specific translocation, we have further reviewed the relevant literature.

## Case presentation

On August 12, 2019, a 49-year-old man who presented with fever was referred to the Changhai Hospital (Shanghai, China). The peripheral blood counts of this patient were as follows: white blood cells, 34 × 10^9^/L (with 26% blasts); hemoglobin, 74 g/L; and platelets, 92 × 10^9^/L. The bone marrow (BM) aspirate showed that 22.5% of blast cells were positive for peroxidase staining. Immunophenotypic analysis using multiparameter flow cytometry revealed that the blast cells were myeloperoxidase^+^, cluster of differentiation (CD)13^+^, CD33^+^, CD123^+^, CD34^+^, CD117^+^, CD38^+^, CD11c^+^, CD64^+^, CD14^+^, CD11b^+^, human leukocyte antigen (HLA)–antigen D related^–^, cytoplasmic CD79a^–^, CD7^–^, cCD3^–^, CD19^–^, CD4^–^, CD10^–^, CD15^–^, CD56^–^, CD2^–^, and CD16^–^. The antibodies against CD7, CD19, CD13, CD10, CD14, CD15, CD123, CD38, and CD13 were purchased from BD Biosciences (San Jose, CA, USA), and other antibodies were purchased from Beckman Coulter (Brea, CA, USA). A total of 1 mL freshly isolated whole BM aspirate was collected, of which 400 µL was stained with monoclonal antibodies for 15 min at room temperature. Following red blood cell lysis, BM cells were washed, collected, and analyzed following the manufacturer’s instructions using a FACSAria II instrument (BD Biosciences).

**Fig. 1:**
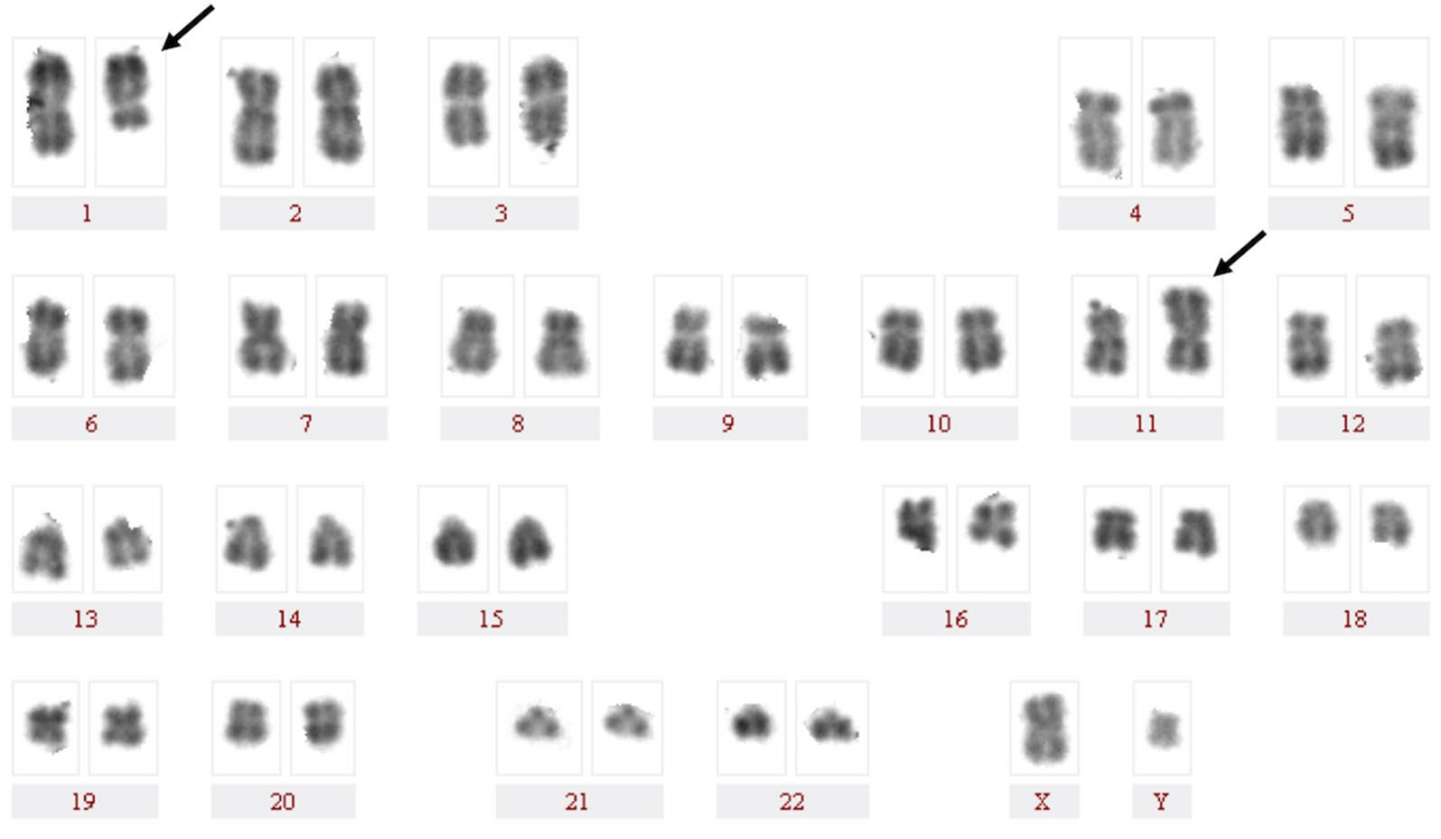
R-Banded karyogram at diagnosis of the patient. Arrowa indicate t(1;11)(q23;p15) chromosomal abnormalities

**Fig. 2:**
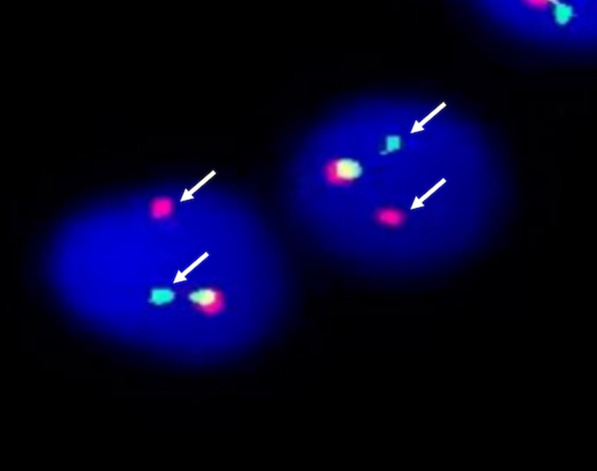
FISH analysis of the patient. FISH analysis by use of the dual-color break-apart probes that showed the typical split signal pattern: split 3'-end (green) and 5'-end (red) probe signals indicated by arrows with a single normal unsplit red-green signal pair (yellow)

**Fig. 3:**
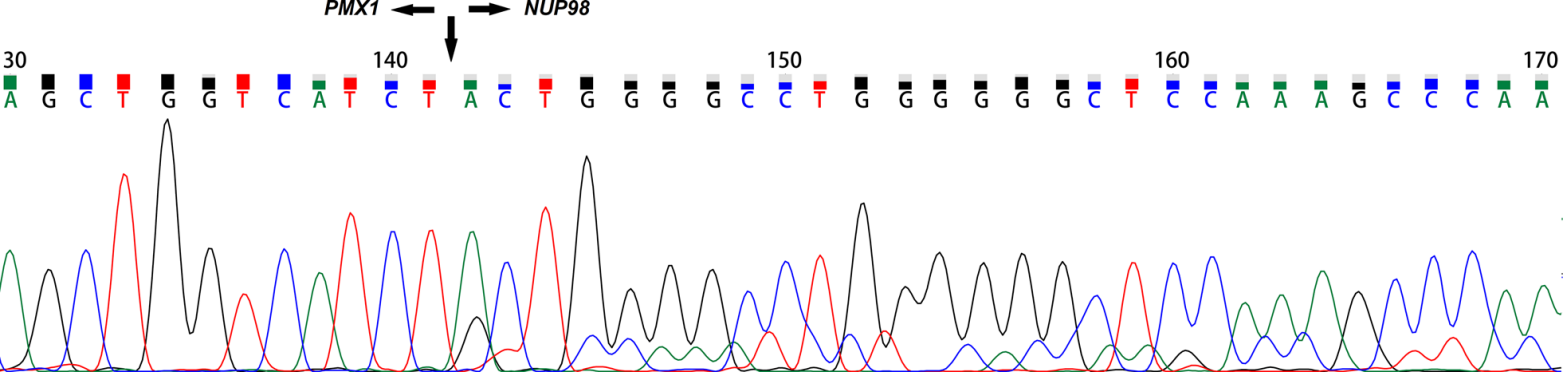
Nucleotide sequences of *NUP98-PMX1* fusions. Arrows indicate the fusion points

The karyotype of the patient was 46,XY,t(1;11)(q23;p15)[20] (Fig. 1). Fluorescence in situ hybridization (FISH) analysis using dual-color break-apart probes was performed with Isis Software (MetaSystems, Germany). Hybridized chromosome slides were analysed using an epifluorescence microscope Axio imager A2 (Carl Zeiss, Germany). The result showed the typical split signal pattern as split 3'-end (green) and 5'-end (red) probe signals along with a single normal unsplit red-green signal pair (yellow). This indicated that *NUP98* was disrupted as a result of translocation (Fig. 2). Then we performed reverse transcription-polymerase chain reaction (RT-PCR) analysis of the BM cells. The sequences of primers were referred to the previously reported of Nakamura et al. [5]. RNA was extracted from the BM cells with the Trizol reagent (15596026, Thermo Fisher Scientific, Inc., Waltham, MA, USA). PCR was performed on the Agilent SureCycler 8800 (Agilent Technology Inc., Santa Clara, CA, USA) according to the manufacturer's instructions. Sequence analysis of this product confirmed the *NUP98-PMX1* fusion transcript (Fig. 3). The sequence datasets are available in the Additional file 1. Next-generation sequencing analysis revealed FMS-like tyrosine kinase-3 (*FLT3*)-internal tandem duplication (ITD) and neuroblastoma RAS (*NRAS*) mutations. The sequence datasets are available in the Additional file 2. Therefore, the patient was diagnosed with de novo AML with the *NUP98-PMX1* fusion gene.

The patient received idarubicin and cytarabine (idarubicin, 8 mg/m^2^/day on days 1–3; cytarabine, 100 mg/m^2^/day on days 1–7) as induction chemotherapy. After 3 weeks, the BM aspirate showed complete remission. The second RT-PCR result for the *NUP98-PMX1* fusion gene was negative, and the second mutation analysis showed that *FLT3*-ITD and *NRAS* mutations were cleared. Subsequently, the patient received three cycles of high-dose Ara-c (3 g/m^2^ q12 h on days 1–3) as consolidation chemotherapy. During chemotherapy, BM aspiration was performed before each consolidation therapy, and the *NUP98-PMX1* fusion gene was invariably negative. Then, the patient underwent a partially matched (HLA-DP locus mismatch) unrelated allogeneic hematopoietic stem cell transplantation (HSCT) along with myeloablative conditioning with busulfan (3.2 mg/kg/day on days –8 to –6), cyclophosphamide (1.8 g/m^2^/day on days –5 to –4), and anti-thymoglobulin (8 mg/kg dose divided over 3 days). The patient had received mononuclear and CD34^+^ cells (6.5 × 10^8^/kg and 2.5 × 10^6^/kg, respectively). The prophylaxis regimen for acute graft versus host disease consisted of cyclosporine A, mycophenolate mofetil, and short-term methotrexate. Engraftment was confirmed on day 14 after HSCT by the peripheral absolute neutrophil count of more than 0.5 × 10^9^/L for 3 consecutive days and platelet count of more than 20 × 10^9^/L for 7 consecutive days. The follow-up period ended on September 30, 2020 (6 months after HSCT), and the patient exhibited no recurrence or transplantation-related complications. The study was conducted according to the guidelines of the Declaration of Helsinki, and the patient provided an informed consent.

## Discussion and conclusion

*NUP98* gene fusions interfere with the expression of downstream transcription genes and participate in cell proliferation, differentiation, and nucleocytoplasmic exports, thereby promoting myeloid leukemogenesis. Moreover, *NUP98* gene fusions co-occur with a set of additional mutations, including *FLT3*-ITD and other events contributing to increased cell proliferation [9–14]. Although translocations with *NUP98* involvement are rare, they are recurrent in different types of leukemia. Generally, the frequency of *NUP98* gene fusion has been reported to be less than 5% in adult AML [13, 15, 16].

The presence of *NUP98* gene fusions defines a high-risk leukemia subset and has been shown to result in remarkably high induction failure and poor survival [9, 10, 13–21]. Notably, patients with AML harboring *NUP98* gene fusions with concomitant *FLT3*-ITD have a worse prognosis than those without genetic aberrations, and the poor outcomes are determined by the interaction between *NUP98* gene fusions and *FLT3*-ITD [10, 12, 22]. Thanasopoulou et al. [23] found that co-expression of *FLT3*-ITD increased cell proliferation and maintained self-renewal ability in a *NUP98* gene fusion-positive mouse model.

Although *NUP98* has a series of functionally diverse partner genes, the most observed *NUP98* fusion partners belong to the *HOX* gene family. The *NUP98-HOXA9* gene, resulting from t(7;11)(p15;p15), is the most common fusion gene. Patients with AML harboring the *NUP98-HOXA9* rearrangement were found to have poorer overall survival (OS) and relapse-free survival (RFS) than those not harboring this rearrangement, even when patients with low-risk karyotypes were excluded (median OS: 13.5 months vs. 20 months, *P* = 0.045; median RFS: 6 months vs. 12 months, *P* = 0.003) [13, 16].

*PMX1* is a member of the class II *HOX* gene family located at 1q23. The function of *PMX1* in the hematopoietic system and leukemogenesis remains unknown. Moreover, the formation of the *NUP98-PMX1* fusion gene caused by t(1;11)(q23;p15) is rarely reported, and its clinical features and outcomes remain to be clarified. To date, only five cases with *NUP98-PMX1* have been reported (Table 1). *NUP98-PMX1* juxtaposition was confirmed in this patient using RT-PCR and FISH. As shown in Table 1, five of the six patients (including the patient described here) were male. The median age of patients at diagnosis was 50 years (range 42–74 years). Thus, it seems the t(1;11)(q23;p15) occurs at a higher frequency in older male patients, though more cases are needed to solidify this relationship.

The mechanisms of the *NUP98-PMX1* fusion protein underlying leukemogenesis remain unclear. Studies have reported that *NUP98-PMX1* but not *PMX1* has the ability to impair differentiation and promote proliferation of hematopoietic progenitor cells in vitro [24]. Mice transplanted with *NUP98-PMX1*-transduced BM cells has a potent effect on induction of myeloproliferative disease [24, 25]. Moreover, the fusion protein might act as an oncogenic transcription factor. It has been shown that the in-frame fusion of PMX1 HD and the N-terminal GLFG repeat of NUP98 results in strong transcriptional activation and PMX1 HD upregulation [5]. Constitutive expression and alteration of the transcriptional activity of PMX1 HD may substantially contribute to myeloid leukemogenesis. This evidence further supports the involvement of *NUP98-PMX1* in the occurrence and development of myeloid leukemia.

Since *NUP98* gene fusions are now recognized as markers of a high-risk leukemia subset, current treatment paradigms often utilize chemotherapy followed by HSCT during the first complete molecular remission. Our patient with *NUP98-PMX1* and concomitant *FLT3*-ITD achieved molecular remission after induction chemotherapy. Subsequently, he underwent HSCT and was disease-free at the time of the last visit. However, the excellent prognosis of our patient may be due to the relatively short follow-up period.

Since reports of patients carrying the *NUP98-PMX1* fusion gene are limited, it is difficult to deduce any conclusions involving the prognostic significance of this gene. This is the first report of a patient with de novo AML carrying the *NUP98-PMX1* fusion gene, which may contribute to a more comprehensive profile of this genetic rearrangement. In the future, mechanistic studies are needed to investigate the role of the *NUP98-PMX1* fusion gene in leukemia pathogenesis.

## Supplementary Information

**Additional file 1**. Nucleotide sequences of NUP98-PMX1 fusions by Sanger sequencing.

**Additional file 2**. The patient’s genome sequencing datasets.

## Data Availability

All data generated or analysed during this study are included in this published article and its supplementary information files. The details of the variant analysed during the current study are available in the SRA database, under the accession number PRJNA728242.

## Abbreviations

AML: Acute myeloid leukemia
BM: Bone marrow
CD: Cluster of differentiation
FG: Phenylalanine-glycine
FISH: Fluorescence in situ hybridization
FLT3: FMS-like tyrosine kinase-3
GLFG: Glycine-leucine-phenylalanine-glycine
HD: Homeodomain
HLA: Human leukocyte antigen
HOX: Homeobox
HSCT: Hematopoietic stem cell transplantation
ITD: Internal tandem duplication
NRAS: Neuroblastoma RAS
NUP98: Nucleoporin 98
OS: Overall survival
PMX1: Paired related homeobox 1
RFS: Relapse-free survival
RT-PCR: Reverse transcription-polymerase chain reaction

## References

[1] Radu A, Moore MS, Blobel G (1995). The peptide repeat domain of nucleoporin Nup98 functions as a docking site in transport across the nuclear pore complex. Cell.

[2] Rosenblum JS, Blobel G (1999). Autoproteolysis in nucleoporin biogenesis. Proc Natl Acad Sci.

[3] Pritchard CE, Fornerod M, Kasper LH (1999). RAE1 is a shuttling mRNA export factor that binds to a GLEBS-like NUP98 motif at the nuclear pore complex through multiple domains. J Cell Biol.

[4] Michmerhuizen NL, Klco JM, Mullighan CG (2020). Mechanistic insights and potential therapeutic targets for NUP98-rearranged hematologic malignancies. Blood.

[5] Nakamura T, Yamazaki Y, Hatano Y (1999). NUP98 is fused to PMX1 homeobox gene in human acute myelogenous leukemia with chromosome translocation t(1;11)(q23;p15). Blood.

[6] Zhang L, Alsabeh R, Mecucci C (2007). Rare t(1;11)(q23;p15) in therapy-related myelodysplastic syndrome evolving into acute myelomonocytic leukemia: a case report and review of the literature. Cancer Genet Cytogenet.

[7] Kobzev YN, Martinez-Climent J, Lee S (2004). Analysis of translocations that involve theNUP98 gene in patients with 11p15 chromosomal rearrangements. Genes Chromosom Cancer.

[8] Bai XT, Gu BW, Yin T (2006). Trans-repressive effect of NUP98-PMX1 on PMX1-regulated c-FOS gene through recruitment of histone deacetylase 1 by FG repeats. Cancer Res.

[9] Niktoreh N, Walter C, Zimmermann M (2019). Mutated WT1, FLT3-ITD, and NUP98-NSD1 fusion in various combinations define a poor prognostic group in pediatric acute myeloid leukemia. J Oncol.

[10] Bisio V, Zampini M, Tregnago C (2017). NUP98-fusion transcripts characterize different biological entities within acute myeloid leukemia: a report from the AIEOP-AML group. Leukemia.

[11] Bolouri H, Farrar JE, Triche T (2019). Publisher correction: the molecular landscape of pediatric acute myeloid leukemia reveals recurrent structural alterations and age-specific mutational interactions. Nat Med.

[12] Ostronoff F, Othus M, Gerbing RB (2014). NUP98/NSD1 and FLT3/ITD coexpression is more prevalent in younger AML patients and leads to induction failure: a COG and SWOG report. Blood.

[13] Chou WC, Chen CY, Hou HA (2009). Acute myeloid leukemia bearing t(7;11)(p15;p15) is a distinct cytogenetic entity with poor outcome and a distinct mutation profile: comparative analysis of 493 adult patients. Leukemia.

[14] Burillo-Sanz S, Morales-Camacho RM, Caballero-Velazquez T (2016). NUP98-HOXA9 bearing therapy-related myeloid neoplasm involves myeloid-committed cell and induces HOXA5, EVI1, FLT3, and MEIS1 expression. Int J Lab Hematol.

[15] Hollink IH, van den Heuvel-Eibrink MM, Arentsen-Peters ST (2011). NUP98/NSD1 characterizes a novel poor prognostic group in acute myeloid leukemia with a distinct HOX gene expression pattern. Blood.

[16] Wei S, Wang S, Qiu S (2013). Clinical and laboratory studies of 17 patients with acute myeloid leukemia harboring t(7;11)(p15;p15) translocation. Leuk Res.

[17] Struski S, Lagarde S, Bories P (2017). NUP98 is rearranged in 3.8% of pediatric AML forming a clinical and molecular homogenous group with a poor prognosis. Leukemia.

[18] Miyamura T, Moritake H, Nakayama H (2019). Clinical and biological features of paediatric acute myeloid leukaemia (AML) with primary induction failure in the Japanese Paediatric Leukaemia/Lymphoma Study Group AML-05 study. Br J Haematol.

[19] Yang J, Lyu X, Zhu X (2017). Chromosome t(7;11)(p15;p15) translocation in acute myeloid leukemia coexisting with multilineage dyspoiesis and mutations in NRAS and WT1: a case report and literature review. Oncol Lett.

[20] de Rooij JD, Masetti R, van den Heuvel-Eibrink MM (2016). Recurrent abnormalities can be used for risk group stratification in pediatric AMKL: a retrospective intergroup study. Blood.

[21] Marceau-Renaut A, Duployez N, Ducourneau B (2018). Molecular profiling defines distinct prognostic subgroups in childhood AML: a report from the French ELAM02 study group. HemaSphere.

[22] Shimada A, Iijima-Yamashita Y, Tawa A (2018). Risk-stratified therapy for children with FLT3-ITD-positive acute myeloid leukemia: results from the JPLSG AML-05 study. Int J Hematol.

[23] Thanasopoulou A, Tzankov A, Schwaller J (2014). Potent co-operation between the NUP98-NSD1 fusion and the FLT3-ITD mutation in acute myeloid leukemia induction. Haematologica.

[24] Hirose K, Abramovich C, Argiropoulos B (2008). Leukemogenic properties of NUP98-PMX1 are linked to NUP98 and homeodomain sequence functions but not to binding properties of PMX1 to serum response factor. Oncogene.

[25] Wang Y, Gu MM, Tan Y (2004). Development of human myeloid leukemia-like phenotype in NUP98-PMX1 transgenic mice. Zhonghua Xue Ye Xue Za Zhi.

