# Supplementary material for: First case report of a NUP98-PMX1 rearrangement in de novo acute myeloid leukemia and literature review
Source: BMC Med Genomics. 2021 May 17;14:130. doi: 10.1186/s12920-021-00979-y (PMC8130325; doi:10.1186/s12920-021-00979-y)

**Supplementary file 1**

**First case report of a *NUP98-PMX1* rearrangement in *de novo* acute myeloid leukemia and literature review**

Weijia Fu^#^, MD, Aijie Huang^#^, MD, Hui Cheng, MD, Yanrong Luo, MD, Lei Gao, MD, Gusheng Tang, MD, Jianmin Yang, MD, Jianmin Wang, MD, and Xiong Ni*, MD

Department of Hematology, Institute of Hematology, Changhai Hospital, Shanghai 200433, China

^#^ W.F. and A.H. contributed equally to this work.

* Corresponding author

Supplementary Figure 1. Nucleotide sequences of NUP98-PMX1 fusions by Sanger sequencing (335 bases in 13109 scans).


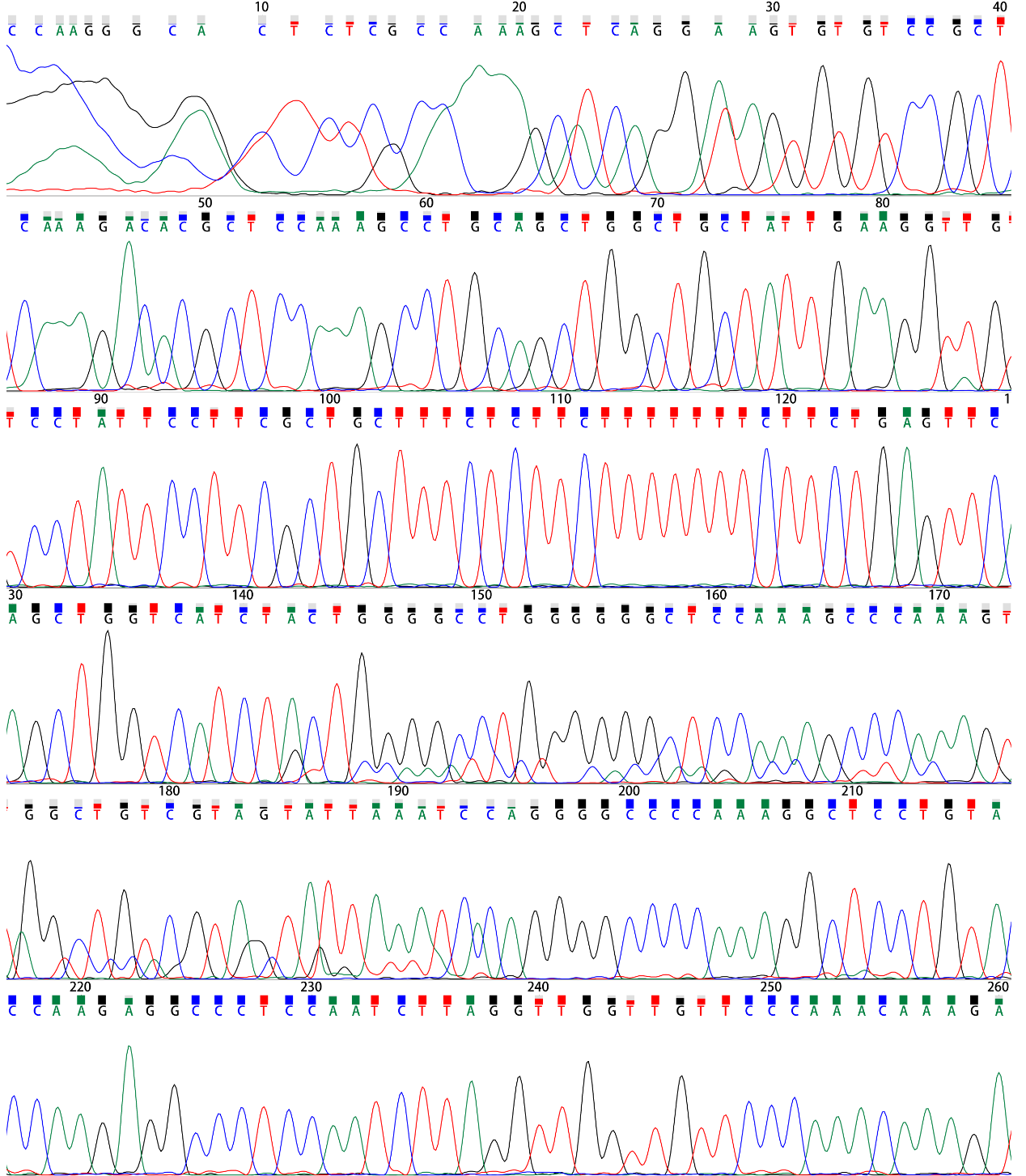


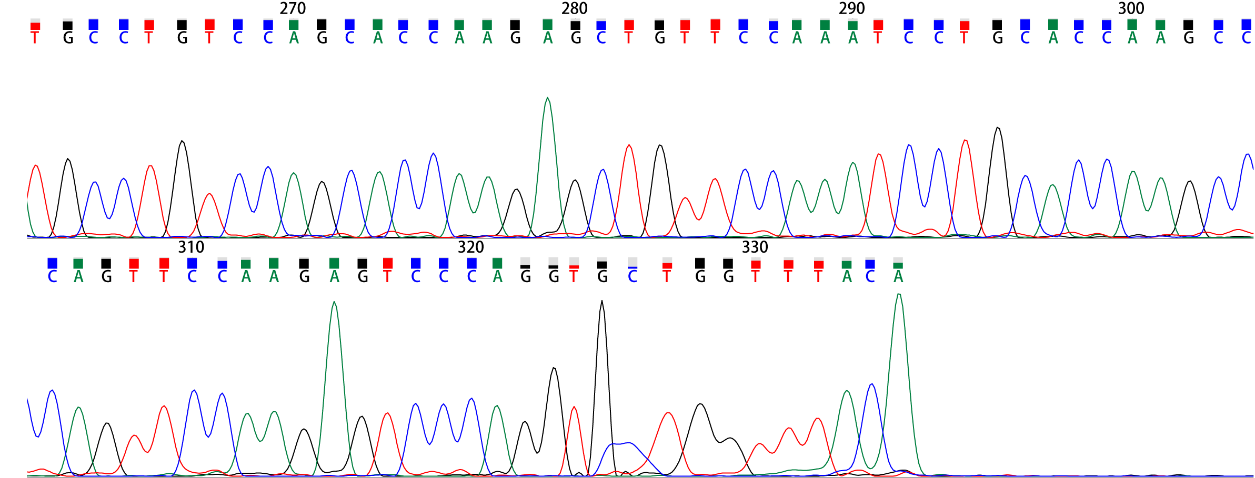

Supplement: Supplementary file 1 — Additional file 1. Nucleotide sequences of NUP98-PMX1 fusions by Sanger sequencing. [file 12920_2021_979_MOESM1_ESM.docx]
